# Vitamin C Intake and Cancers: An Umbrella Review

**DOI:** 10.3389/fnut.2021.812394

**Published:** 2022-01-20

**Authors:** Zeyu Chen, Yin Huang, Dehong Cao, Shi Qiu, Bo Chen, Jin Li, Yige Bao, Qiang Wei, Ping Han, Liangren Liu

**Affiliations:** ^1^Department of Urology, Institute of Urology, National Clinical Research Center for Geriatrics, West China Hospital, Sichuan University, Chengdu, China; ^2^West China School of Clinical Medicine, Sichuan University, Chengdu, China

**Keywords:** vitamin C, cancer incidence, cancer outcome, umbrella review, ascorbate acid

## Abstract

Based on the existing systematic reviews and meta-analyses, we conducted this umbrella review aiming at evaluating the quality of evidence, validity and biases of the relationship between vitamin C (VC) intake and incidence and outcomes of multiple cancers. We identified 22 cancer outcomes within 3,562 articles. VC consumption was associated with lower incidence of bladder cancer, breast cancer, cervical tumors, endometrial cancer, esophageal cancer, gastric cancer, glioma, lung cancer, pancreatic cancer, prostate cancer, renal cell cancer, and total cancer occurrence. VC intake was also related to decreased risk of breast cancer prognosis (recurrence, cancer-specific mortality, and all-cause mortality).

## Introduction

With the aging and growth of human beings and also changes in the prevalence and distribution of cancer risk factors (some of them are socioeconomic development related), the burden of cancer incidence and mortality is rapidly growing (1). Cancer has become the first or second leading cause of death (1, 2). As a result of the rising incidence of cancer, more and more people have been suffering from cancers physically and socioeconomically, and finding anticancer agents has become an urgent need.

Vitamin C (VC), as a wound improving and infectious reducing agent, has been known and used for decades (3). It is a water-soluble vitamin that plays essential roles in antioxidant procedure, collagen biosynthesis, carnitine and catecholamine metabolism, and dietary iron absorption (4).

VC could not be synthesized by the human body, and people could only obtain VC through foods or drugs (4). It is one of the most common micronutrient through citrus fruits, berries, tomatoes, potatoes, and green leafy vegetables (5). The anticancer effect of VC was first reported in 1959 (6) and further demonstrated in 1970's that VC could reduce cancer cell proliferation through direct incorporation into a hyaluronidase inhibitor complex (7).

The association between VC and various cancer outcomes has been evaluated in a large amount of cohort, case-control, and randomized controlled studies. Multiple systematic reviews and meta-analyses summarized results from these studies. However, a comprehensive overview into the correlation between VC and cancers is still in deficiency. Therefore, we are conducting this study aiming at making a comprehensive review of the association between VC and cancer outcomes reported in systematic reviews and meta-analyses and assessing the validity and also level of existing evidence.

## Materials and Methods

### Umbrella Review Method

We comprehensively searched and evaluated published evidence on the association between VC intake and multiple cancer outcomes from a large number of systematic reviews and meta-analyses (8, 9). Systematic reviews without meta-analyses were excluded because they failed to offer quantitative assessment of association between VC intake and cancer outcomes (10).

### Literature Search

We searched systematic reviews and meta-analyses of observational studies and interventional studies from MEDLINE, Embase, and Cochrane Database of Systematic Reviews and Web of Science from the inception to April 2021. The searching strategy was VC AND systematic review OR meta-analyses. The SIGN guidance for systematic reviews and meta-analyses was used for literature search (11, 12). Two investigators (ZYC and YH) screened the titles and abstracts independently and selected eligible articles through full-text review. Any discrepancies in selecting articles between the two researchers were resolved by a third investigator (DHC). The references cited in all eligible articles were also manually searched.

### Eligibility Criteria

Meta-analyses and systematic reviews with meta-analysis of observational (cohort and case-control) and interventional studies (randomized and nonrandomized controlled trials) evaluating VC intake and cancer outcomes in humans were included regardless of the race, gender, country, or region of participants. If two or more cancer outcomes existed in a single article, data of each outcome would be extracted separately. If one cancer outcome was assessed by more than one studies, article with the largest number of participants would be included. Furthermore, articles reporting VC intake with therapeutic utilities were also excluded only if nontherapeutic intake was also reported. Articles written in languages other than English and not involving humans were also excluded.

### Data Extraction

The following data were extracted by ZYC and YH independently from eligible studies: (1) name of the first author, (2) journal, (3) year of publication, (4) category of exposure (dietary VC intake, supplementary intake, and unknown), (5) outcome, (6) number of included studies, (7) number of participants in each study, (8) study design (case-control, cohort, randomized controlled trial (RCT) and nonrandomized controlled trial (NRCT), (9) follow-up time, (10) type of comparisons (highest vs. lowest, any vs. never, and increment or reduction of any dose of VC), (11) the estimated summary effect (RR, relative risk; OR, odds ratio), and corresponding 95% confidence intervals (CIs).

### Assessment of Methodological Quality of Included Studies and Quality of Evidence

Methodological quality of included articles was evaluated following the AMSTAR items, and this is a reliable strategy in assessing the quality of systematic reviews and meta-analyses (10, 13). The Grading of Recommendations, Assessment, Development, and Evaluation (GRADE) was used for assessing the strength of evidence for each outcome presented in the umbrella review and classifying evidence into “high,” “moderate,” “low,” and “very low” quality to making recommendations (14).

### Data Analysis

We extracted data of VC consumption and cancer outcomes, and estimated summary effect with 95% CI reported in each meta-analysis if available (10, 15). If both cohort studies and case-control studies existed in one article, data would be extracted separately if possible. *I*^2^ statistic and Cochran's *Q* test were used to estimate the heterogeneity between studies. Estimation of publication bias in each meta-analysis was presented as result of Egger's regression test (16). Dose-response effects of VC intake on cancer outcomes were also presented if available. *p* <0.10 was regarded as significant for Egger's test and heterogeneity. In addition, *p* < 0.05 was regarded as significant for other tests. Evidence synthesis was performed *via* Review Manager 5.3 version (Cochrane Collaboration, Oxford, UK).

## Results

### Characteristics of Included Meta-Analyses

**Figure 1:**
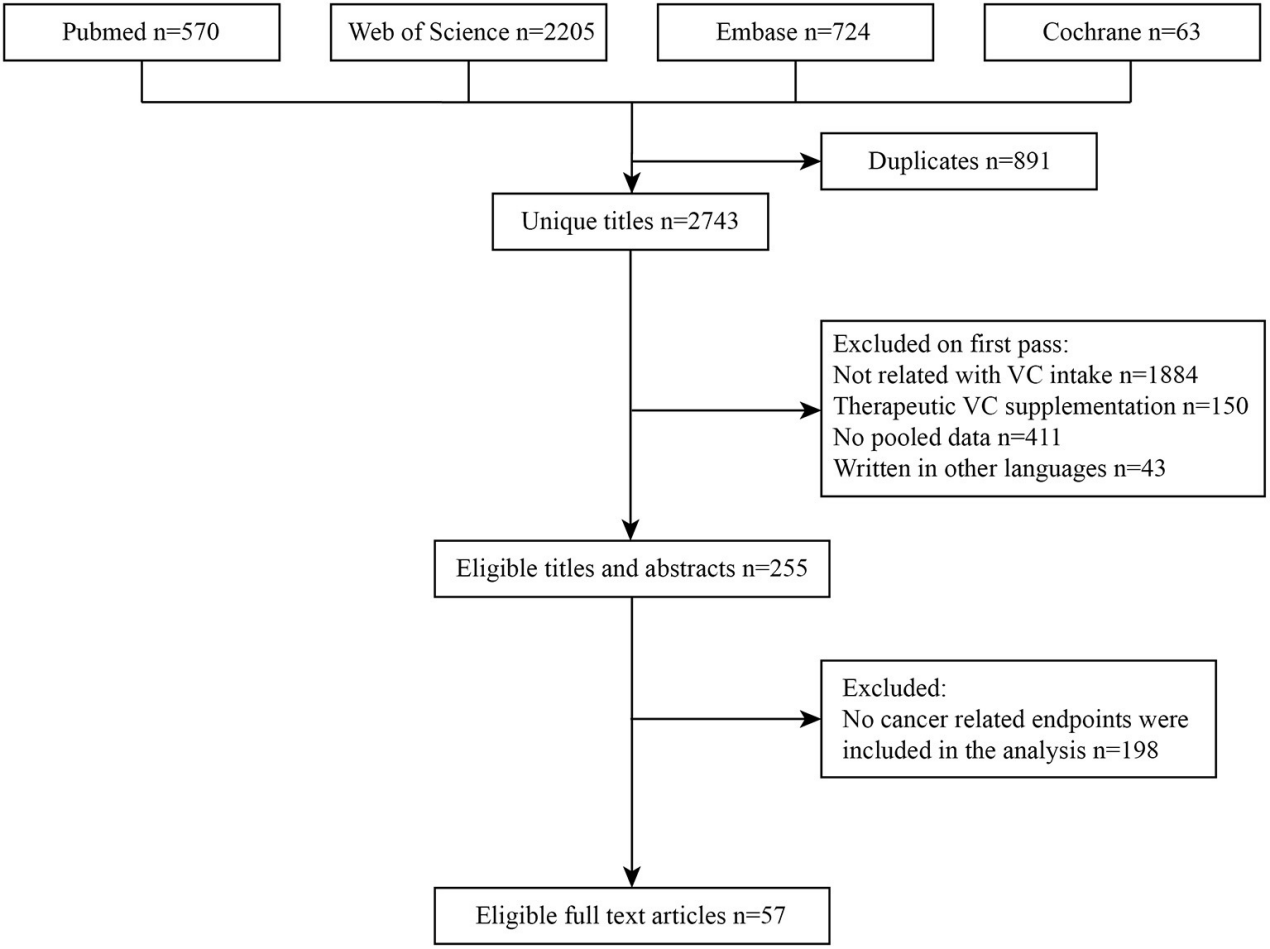
Flowchart of the systematic search and selection process.

The detailed process of literature search and selection was presented in Figure 1. We searched 3,562 articles and finally identified 57 meta-analyses according to our inclusion and exclusion criteria. Nineteen cancer-related outcomes related to VC intake were extracted from all eligible studies. The associations of VC intake with multiple cancer outcomes were presented in Table 1.

**Table 1:** Associations between VC intake and cancer outcomes.

**Outcome**	**Ref. no**.	**Categories**	**No. of cases/total**	**MA metrics**	**Estimates**	**95% CI**	**No. of studies**	**Cohort/Case-control**	**VC intake dose**	**Effects model**	***I^**2**^*; Q test *p*-value**	**Egger test *p-*value**
**Significant associations**												
Bladder cancer risk	17	Diet	5,765/292,002	RR	0.84	0.73–0.98	14	7/7	100 mg/d^*^	Random	47.5%; 0.025	0.28
Breast cancer risk	18	Diet	25,878/1,161,698	RR	0.89	0.82–0.96	31	15/16	>350 mg/d^#^	Random	79.3%; <0.001	0.006
Breast cancer-specific mortality	18	NA	1,813/17,077	HR	0.78	0.69–0.88	6		≥208 mg/d^#^	Random	2.6%; 0.4	NA
Breast cancer recurrence	18	NA	907/7,141	HR	0.81	0.67–0.99	2		NA	Random	0.0%; 0.955	NA
Breast cancer all-cause mortality	18	NA	3,733/26,347	HR	0.82	0.74–0.91	7		>92.5 mg/d^#^	Random	16.6%; 0.303	NA
Cervical neoplasm risk	27	NA	3,761/304,769	OR	0.58	0.44–0.75	12	1/11	≥280 mg/d^#^	Random	68.8%; 0.000	0.009
Endometrial cancer risk	19	Diet	4192/9633	OR	0.85	0.73-0.98	11	1/10	>183 mg/d^#^	Random	66.1%; 0.003	NA
Esophageal cancer risk	22	Diet	3,955/7,063	OR	0.58	0.49–0.60	20	1/19	50 mg/d^*^	Random	56%; 0.001	0.26
Gastric cancer risk	23	Diet	4,101/262,469	RR	0.66	0.59–0.73	37	3/34	100 mg/d^*^	Random	4%; 0.4	0.254
Glioma risk	28	NA	3,409/549,674	RR	0.86	0.75–0.99	15	2/13	NA	Random	12.6%; 0.312	0.487
Lung cancer risk	29	NA	9,028/578,402	RR	0.83	0.73–0.94	21	11/10	100 mg/d^*^	Random	57.8%; 0.001	0.654
Pancreatic cancer risk	24	NA	5426/776039	RR	0.7	0.61-0.81	17	4/13	NA	Random	42.3%; 0.034	0.414
Prostate cancer risk	20	Diet	15,926/87732	RR	0.89	0.83–0.94	18	6/12	>240 mg/d^#^	Random	39.4%; 0.045	<0.05
Renal cell cancer risk	21	NA	5,182/270,425	RR	0.78	0.69–0.87	10	3/7	>585 mg/d^#^	Random	0.0%; 0.655	0.515
Total cancer risk	30	Diet	7,068/181,318	RR	0.87	0.78–0.95	7	7/0	200 mg/d^#^	Random	17.7%; 0.91	0.3
**Nonsignificant associations**												
Bladder cancer risk	17	supplement	3,331/1,199,984	RR	0.87	0.69–1.11	9	6/3	100 mg/d^*^	Random	64.9%; 0.004	0.002
		supplement+diet	2021/194443	RR	0.86	0.67-1.10	8	3/5	100 mg/d^*^	Random	52.5%; 0.040	0.03
Breast cancer risk	18	Supplement	15,920/511,353	RR	1.02	0.94–1.10	13	9/4	>1,000 mg/d^#^	Random	36.4%; 0.092	0.006
Colon Cancer risk	25	Diet	908/104,348	RR	0.87	0.63–1.21	3	3/0	500 mg/d^#^	Random	77.4%; 0.01	NA
Colorectal cancer risk	26	NA	6,542/100,3710	RR	0.92	0.80–1.06	13	13/0	NA	Random	34.9%; 0.94	0.94
non-Hodgkin lymphoma risk	31	Supplement	2,886/120,4336	RR	1	0.90–1.12	8	8/0	≥750 mg/d^#^	Random	0.0%; 0.523	NA

# *Maximum dose of VC intake*.

* *Increment dose of VC intake*.

### Associations Between VC Intake and Cancers of Urogenital System

In the estimation of highest VC intake vs. lowest, significant inverse associations were seen in VC intake and incidence of several cancers of the urogenital system: bladder cancer (source of VC intake: dietary, RR 0.84, 95% CI 0.73–0.98) (17), breast cancer (source of VC intake: dietary, RR 0.89, 95% CI 0.82–0.96) (18), endometrial cancer (source of VC intake: dietary, RR 0.85, 95% CI 0.73–0.98) (19), prostate cancer (source of VC intake: dietary, RR 0.89, 95% CI 0.83–0.94) (20), and renal cell carcinoma (source of VC intake: dietary, RR 0.78, 95% CI 0.69–0.87) (21). Additionally, VC was also related to decreased risk of breast cancer-specific mortality (source of VC intake: unknown, HR 0.78, 95% CI 0.69–0.88), breast cancer recurrence (source of VC intake: unknown, HR 0.81, 95% CI 0.67–0.99), and breast cancer all-cause mortality (source of VC intake: unknown, HR 0.82, 95% CI 0.74–0.91) (18). Nonsignificant association was detected in VC intake and risk of bladder cancer (supplementary intake/supplementary+dietary intake) (17) and breast cancer (supplementary intake) (18).

When estimating the dose-response effect of these associations, we found that every 50 mg/1,000 kcal increment of VC intake was related to a 15% (95% CI 0.73–0.98) decrease in the risk of endometrial carcinoma, and a 150 mg/day increment of dietary VC intake was related to 9% (95% CI 0.84–0.98) lower incidence of prostate cancer (20).

In subgroup analysis, we found that VC intake was significantly related to the risk of breast cancer in case-control studies (RR 0.74, 95% CI 0.65–0.84) and in studies of Asia (RR 0.62, 95% CI 0.48–0.80) (18); risk of prostate cancer in case-control studies (RR 0.80, 95% CI 0.71–0.89), cohort studies (RR 0.92, 95% CI 0.86–0.99), and studies of United states (RR 0.89, 95% CI 0.83–0.95) (20); risk of renal cell cancer in Americans (RR 0.81, 95% CI 0.67–0.96) and Europeans (RR 0.76, 95% CI 0.65–0.88) and also case-control studies (RR 0.75, 95% CI 0.66–0.86) (21).

### Associations Between VC Intake and Cancers of Digestive System

Comparing the relationship between highest vs. lowest intake of ascorbic acid and the incidence of digestive system cancers, we found that highest intake of VC was related to the reduced risk of esophageal cancer (source of VC intake: dietary, RR 0.58, 95% CI 0.49–0.60) (22), gastric cancer (source of VC intake: unknown, RR 0.66, 95% CI 0.59–0.73) (23), and pancreatic cancer (source of VC intake: unknown, RR 0.70, 95% CI 0.61–0.81) (24) compared with the lowest income. Nonsignificant association was found between VC intake and incidence of colon cancer (25) and also colorectal cancer (26). It was shown by dose-response analysis that every 50 mg/day increment of VC intake was related to a 13% decrease in esophageal cancer risk (95% CI 0.80–0.93) (22), and every 100 mg/day increment of VC intake was associated with a 26% reduce in gastric cancer risk (95% CI 0.69–0.79) (23).

When subgroup analysis was conducted, significant associations of VC intake and pancreatic cancer risk were found in case-control studies (RR 0.65, 95% CI 0.55–0.76), cohort studies (RR 0.827, 95% CI 0.65–0.99), Caucasian (RR 0.74, 95% CI 0.63–0.88), and Asian (RR 0.455, 95% CI 0.28–0.75) and also mixed population (RR 0.68, 95% CI 0.51–0.90) (24).

### Associations Between VC Intake and Cancers of Nervous System

In the estimation of highest VC intake vs. lowest, we detected an inverse association of the risks of nervous system neoplasms: incidence of cervical neoplasms (source of VC intake: unknown, RR 0.58, 95% CI 0.44–0.75) (27) and glioma (source of VC intake: unknown, RR 0.86, 95% CI 0.75–0.99) (28) was decreased by 42% and 14%, respectively. Dose-response analysis showed that every 50 mg/day increment of VC intake was related to 8% decrease in the risk of cervical neoplasm (95% CI 0.89–0.94) (27). Furthermore, significant positive associations were also found in the American population (RRs 0.85, 95% CI 0.73–0.98) and case-control studies (RR 0.80, 95% CI 0.69–0.93) in VC intake and glioma risk (28).

In subgroup analyses of cervical neoplasms, significant effect was observed in histological subtypes. VC intake was associated with reduced risk of invasive cervical carcinoma (OR 0.64, 95% CI 0.57–0.77), considering stratification of geographic area, and studies from Europe and America showed that VC intake had a significant correlation with the risk of cervical neoplasm (OR 0.58, 95% CI 0.43–0.77). When stratified by study design, inverse association of VC intake and risk of cervical neoplasm was revealed in population-based case-control (OR 0.56, 95% CI 0.42–0.75) and hospital-based case-control studies (OR 0.51, 95% CI 0.35–0.76). When stratified by dose of VC intake, all the investigated concentrations of VC intake were significantly correlated with reduced incidence of cervical neoplasm (<50 mg/day: OR 0.58, 95% CI 0.36–0.94; 50–100 mg/day: OR 0.58, 95% CI 0.38–0.89; >100 mg/day: OR 0.63, 95% CI 0.49–0.82) (27).

### Associations Between VC Intake and Other Cancers

We also detected significant associations in ascorbic acid intake with incidence of lung cancer (source of VC intake: unknown, RR 0.83, 95% CI 0.73–0.94) (29) and total cancer (source of VC intake: dietary, RR 0.87, 95% CI 0.78–0.95) (30) comparing highest intake with lowest. Intake of VC was not related to risk of non-Hodgkin lymphoma (source of VC intake: supplementary, RR 1.00, 95% CI 0.90–1.12) (31). When estimating the dose-response effect of VC on these cancers, the results of pooled estimations showed 7% decrease in incidence of lung cancer (95% CI 0.88–0.98) (29) and total cancer (95% CI 0.87–0.99) (30) with 100 mg/day increment of VC intake.

Results from subgroup analyses showed that significant relationship between VC and total cancer incidence was observed in studies in Asia (RR 0.91, 95% CI 0.84–0.99), study with cases more than 1,000 (RR 0.93, 95% CI 0.89–0.97), study quality of 7–9 points (RR 0.93, 95% CI 0.87–0.99) (30).

### Heterogeneity and Publication Bias of Included Studies

Twelve meta-analyses among all 19 included articles showed *Q*-test *p* <0.10. Nine meta-analyses reported low level of heterogeneity (*I*^2^ <25%).

Nine studies of included studies were reported to have significant publication bias, whereas this was not detected in five studies.

### AMSTAR and GRADE Evaluation of Included Studies

AMSTAR scores were estimated in our umbrella review, ranging from 4 to 8 points (median 7, interquartile range 6–7). Supplementary Table 1 shows the detailed AMSTAR scores for each outcome. Evidence of colorectal cancer and total cancer incidence showed “high” quality according to the GRADE classification, and “low” quality was observed in bladder cancer risk (supplementary+dietary/supplementary), breast cancer risk (dietary), cervical neoplasm risk, pancreatic cancer risk, and prostate cancer risk, and the others were classified as “moderate” quality. Detailed information of GRADE scores for each outcome is presented in Supplementary Table 2.

## Discussion

The anticancer phenomenon of VC has been reported by a large number of population-based studies and pooled by many meta-analysis and systematic reviews. We conducted this umbrella review aiming at investigating the relationship between VC intake and cancers comprehensively. In total, 57 meta-analyses involving 19 unique outcomes of the correlation between ascorbic intake and cancers were included. As per the results of our study, intake of ascorbic acid was related to lower risk of several cancers involving different systems (bladder cancer, breast cancer, cervical neoplasms, endometrial carcinoma, esophageal cancer, gastric cancer, glioma, lung cancer, pancreatic cancer, prostate cancer, and renal cell cancer). Although these results showed the anticancer potential of ascorbic acid, studies into the underlying mechanism of this effect are still ongoing.

As one of the most common antioxidants obtained from fruits and vegetables (32), VC was reported to have both antioxidant and prooxidant effects at low serum concentration and high serum concentrations, respectively (33–37). Pauling et al. proposed that many patients with malignant neoplasms need VC supplementation in 1979 (38). He also indicated that the prevention of cancer development and progress guarantees VC. Ascorbate involved in a variety of chemical and physical procedures against carcinogens. Increased intake of ascorbic acid could bring measurable benefits in prevention and treatment of cancer (38). It was generally demonstrated by previous studies that VC could prevent cancers by reducing oxidative DNA damage and protecting normal tissues from the harmful effect of carcinogens (32, 39–52). These mechanisms might be explained by the following descriptions.

Ascorbate could reduce metal ions such as iron, copper, etc. (53), thus guaranteeing their catalytic activities. This effect could induce several iron-dependent enzymatic processes that played an important role in DNA synthesis and epigenetics (53). A process of posttranslational modification of collagen, proline, and lysine hydroxylase may lead to disruption of connective tissue function.

Another important process was posttranslationally regulate the level of hypoxia-inducible factor-1 (HIF-1, which regulates transcription of multiple genes related to cancer biology: cell immortality, angiogenesis, and chemo- or radiotherapy resistance) through Fe2+/2-oxoglutarate (2OG)-dioxygenase-dependent pathway, which was also ascorbate dependent (53, 54). Consumption of ascorbate acid could reduce the activation of HIF-1 (53, 54). Level of HIF-1 cell relies on the concentration of oxygen, and increase of HIF-1 level could be regarded as a result of oncogene activation and change of ascorbate availability which could modulate the activity of hydroxylases (53). VC is a epigenetic modulator involved in the reprogramming of hydroxylase cells and ten-eleven translocation (TET) proteins (55, 56). Three members were included in the TET family: TET 1, TET 2, and TET 3, which belong to alpha-ketoglutaric acid and Fe (^2+^)-dependent dioxygenase superfamily (57, 58). TET proteins played an important role in the regulation of DNA demethylation through converting 5-methylcytosine (5 mC) to 5-hydroxymethylcytosine (5 hmC), 5-formylcytosine (5 fC), and 5-carboxylcytosine (5 caC), which was iron (Fe^2+^) and 2-ketoglutarate-dependent (58). Ascorbic acid could regulate the conversion of Fe^3+^ to Fe^2+^ (53), thus playing a catalytic role of this TET-mediated oxidation of 5mC, and provide unique capacity of regulating the dynamics of DNA methylation (59). It was reported in a previous study that addition of VC in the culture of embryonic stem cells could induce DNA demethylation and expression of germline development-related key genes (related to regulation of meiosis and demethylation of germ cells) *via* a TET1-/TET2-dependent way (60). The authors who conducted incubation of germ cells in the VC-deficient environment and who identified 412 genes in total were differentially expressed compared with controls. This result indicated that VC is an important factor in proper expression of germ-line genes and cell development. They then identified 460 different methylated regions across the genome. Hypermethylation occurred in two-thirds of them in the absence of ascorbate. This phenomenon indicates that VC played an important role in DNA demethylation. They detected that most hypermethylated regions are located distantly from transcription start sites and are enriched for transposable elements (LINE1, IAP, and ERVK families), which display methylation gains. Totally, 55 hypermethylated regions are located within 5 kb from transcription start sites which are enriched for germline regulators after VC deficiency (regulators for proper expression of genes in germ cells and meiosis). Their results demonstrated that VC deficiency could induce a genetic mutation with a crucial role in epigenetic reprogramming through a TET-dependent pathway (60).

In another study (59), the authors detected increased level of 5 hmC and 5 fC at a dose-dependent manner by 4.0- to 7.0-fold and 4.6- to 8.9-fold in VC-added (50–500 μM) cultured cells, thus demonstrating that ascorbic acid could significantly promote the level of 5 hmC and 5 fC. They also tested a number of strong reducing chemicals (spermidine, vitamin B1, vitamin E, glutathione, and NADPH, etc.), and no enhancement of TET-mediated oxidation of 5 mC was observed. Together with these results, the authors concluded that ascorbic acid is a unique cofactor of TET dioxygenases. This may be explained by the reducing effect of VC on the process of Fe3+ to Fe2+ for iron recycling during TET-mediated oxidation of 5 mC as described previously. As previously reported, TET played an important role in keeping the methylation balance and stability of genes (57). Activation of cancers could be demonstrated as a process of promoter hypermethylation and suppressor hypomethylation of genes (61). TET has been proved to be related to progression, invasion, and metastasis in several cancers (acute myeloid leukemia, chronic lymphocytic leukemia, acute lymphoblastic leukemia, breast cancer, cervical cancer, epithelial ovarian carcinoma, colorectal cancer, hepatic cancer, pancreatic cancer, and lung cancer, etc.) (61).

It is commonly known that the prooxidative activity of VC relies mostly on Fe availability. Fe^2+^ (Fe^3+^ reduced by ascorbate) could react easily with oxygen and thus could lead to the formation of reactive oxygen species (ROS) and H_2_O_2_ and generated a highly reactive hydroxyl radical (53). H_2_O_2_ could be used quickly and effectively by the appropriate enzyme systems in normal tissues, and this effect could not be accomplished in cancer cells (62). Researchers also indicated that the activity of enzymes that neutralize oxidative stress, catalase, and superoxide dismutase is inhibited (62). Combining these evidences together, the authors demonstrated that ascorbate has the prooxidative potential in cancer cells with impaired metabolism (53).

Reactive oxygen species played an important role in the signaling of normal cells and it may cause cellular damage and lead to cancer by altering cellular regulatory pathways as previously reported by other studies (40, 41). Inhibition of proliferation and differentiation could be observed in cells with low ROS level, and hyperproliferation was found in cells with high ROS level (63). It was reported that increased ROS level was required for the progression of cancer cells. However, excessive ROS level could lead to cell death (64). Previous studies have reported that ROS generation and redox status were altered in cancer cells, which could be more vulnerable to increased oxidative stress (65). It was also demonstrated that elevated exogenous ROS level above a toxic threshold could overwhelm the antioxidant capacity of these cells (66).VC acts as an electron donor that could reduce level of ROS by oxidizing itself to ascorbyl radical (67). After donating an electron, another electron is also donated by ascorbyl radical and then it oxidizes to dehydroascorbic acid. Both ascorbyl radical and dehydroascorbic acid could be reduced back to the original form as ascorbic acid (68). This stabilized, reducible, and reusable biological characteristics of oxidized ascorbic acid may contribute to the fact that VC is a preferred antioxidant in daily use (69). Upregulated antioxidant systems could induce apoptosis, and ascorbate could initiate this effect by modulating the response to oxidative stress and DNA damage by altering redox signaling (70, 71).

The recommended daily VC intake of the Institute of Medicine is 75 mg/d for adult female and 90 mg/d for adult male (72); however, this recommendation was only for the prevention of VC deficiency and has been doubted for years. A range of 250–400 mg daily VC intake was proposed in 1974 and 1999 by Pauling and Carr et al. (38, 73). The minimal intake of VC in included studies is approximately 100 mg/d, and this value could be expanded to more than 500 mg/d in those studies with statistically significant effect. Most of the dose-response analysis showed a linear relationship between VC intake and cancer incidence. Taking this evidence together, we may recommend a daily VC intake of at least 200 mg.

Notably, this is the first comprehensive evaluation and overview of the existing evidence on the association between VC intake and cancer outcomes. Standard tools were used to assess the methodological quality (AMSTAR) and strength of evidence (GRADE) of those included literature. Furthermore, a low publication bias rate was detected among the included meta-analyses. Although methodological patterns were used properly, selection bias may still exist. To minimize this bias, we have two authors who conducted these jobs with those methods described above to achieve this work.

However, several limitations existed in our study. First, only two of the included studies were classified as high quality according to the GRADE method due to the nature of most meta-analyses being conducted based on observational studies. Second, considering that the most commonly seen resources of VC are fruits and vegetables, people can hardly intake VC as the only antioxidant agent or crucial nutrient in their daily life. Those micronutrients taken together might influence the effect of ascorbic acid on cancers. However, these factors were not assessed in subgroup analyses in the included meta-analyses. Finally, the definition of highest intake and lowest intake was not clearly quantified, thus making it hard to define the effect size of the correlations to a standardized baseline, and dose-response analysis was also conducted in no more than half of included meta-analyses. Considering these shortages of this study, further studies looking into this topic are still needed and should be of better quality.

## Conclusions

After comprehensively review of literatures, we concluded that intake of VC was related to lower risk of multiple cancers of diverse systems. As VC was a commonly seen and easily acquired micronutrient, increase of VC enriched foods was highly recommended. At the same time, we are looking forward to seeing population-based studies of higher quality, and laboratory investigations into the mechanism of the anticancer effect of VC are guaranteed in the future.

## Author Contributions

ZC and YH conducted this research and wrote the paper. LL, PH, and QW designed the study and had primary responsibility for final content. SQ, BC, JL, and YB provided essential materials. DC analyzed data. All authors read and approved the final draft.

## Funding

This study was funded by the National Natural Science Foundation of China (grant number 82000721) and Program from Department of Science and Technology of Sichuan Province (grant number 2020YJ0054).

## Conflict of Interest

The authors declare that the research was conducted in the absence of any commercial or financial relationships that could be construed as a potential conflict of interest.

## Publisher's Note

All claims expressed in this article are solely those of the authors and do not necessarily represent those of their affiliated organizations, or those of the publisher, the editors and the reviewers. Any product that may be evaluated in this article, or claim that may be made by its manufacturer, is not guaranteed or endorsed by the publisher.

## Abbreviations

VC: vitamin C
RR: relative risk
OR: odds ratio
HR: hazard ratio
95% CI: 95% confidence interval
TET: ten-eleven translocation
ROS: reactive oxygen species.

## References

[1] Sung H Ferlay J Siegel RL Laversanne M Soerjomataram I Jemal A. Global Cancer Statistics 2020: GLOBOCAN Estimates of incidence and mortality worldwide for 36 cancers in 185 countries. CA Cancer J Clin. (2021) 71:209–49. 10.3322/caac.21660 33538338

[2] (WHO). WHO. Global Health Estimates 2020: Deaths by Cause, Age, Sex, by Country and by Region, 2000-2019. WHO (2020).

[3] Padayatty SJ Katz A Wang Y Eck P Kwon O Lee JH. Vitamin C as an antioxidant: evaluation of its role in disease prevention. J Am Coll Nutr. (2003) 22:18–35. 10.1080/07315724.2003.10719272 12569111

[4] Abdullah M Jamil RT Attia FN. Vitamin C (Ascorbic Acid). In: StatPearls [Internet]. Treasure Island, FL: StatPearls Publishing (2021). 29763052

[5] Fenech M Amaya I Valpuesta V Botella MA. Vitamin C content in fruits: biosynthesis and regulation. Front Plant Sci. (2018) 9:2006. 10.3389/fpls.2018.02006 30733729 PMC6353827

[6] McCormick WJ. Cancer: a collagen disease, secondary to a nutritional deficiency. Arch Pediatr. (1959) 76:166–71. 13638066

[7] Cameron E Rotman D. Ascorbic acid, cell proliferation, and cancer. Lancet. (1972) 1:542. 10.1016/S0140-6736(72)90215-2 4110043

[8] Aromataris E Fernandez R Godfrey CM Holly C Khalil H Tungpunkom P. Summarizing systematic reviews: methodological development, conduct and reporting of an umbrella review approach. Int J Evid Based Healthc. (2015) 13:132–40. 10.1097/XEB.0000000000000055 26360830

[9] Papatheodorou S. Umbrella reviews: what they are and why we need them. Eur J Epidemiol. (2019) 34:543–6. 10.1007/s10654-019-00505-6 30852716

[10] Poole R Kennedy OJ Roderick P Fallowfield JA Hayes PC Parkes J. Coffee consumption and health: umbrella review of meta-analyses of multiple health outcomes. BMJ. (2017) 359:j5024. 10.1136/bmj.j5024 29167102 PMC5696634

[11] Li N Wu X Zhuang W Xia L Chen Y Wu C. Fish consumption and multiple health outcomes: umbrella review. Trends Food Sci Technol. (2020) 99:273–83. 10.1016/j.tifs.2020.02.03332488249

[12] Righi NC Schuch FB De Nardi AT Pippi CM de Almeida Righi G Puntel GO. Effects of vitamin C on oxidative stress, inflammation, muscle soreness, and strength following acute exercise: meta-analyses of randomized clinical trials. Eur J Nutr. (2020) 59:2827–39. 10.1007/s00394-020-02215-2 32162041

[13] Shea BJ Grimshaw JM Wells GA Boers M Andersson N Hamel C. Development of AMSTAR: a measurement tool to assess the methodological quality of systematic reviews. BMC Med Res Methodol. (2007) 7:1–7. 10.1186/1471-2288-7-10 17302989 PMC1810543

[14] Guyatt G Oxman AD Akl EA Kunz R Vist G Brozek J. GRADE guidelines: 1. Introduction-GRADE evidence profiles and summary of findings tables. J Clin Epidemiol. (2011) 64:383–94. 10.1016/j.jclinepi.2010.04.026 21195583

[15] Li N Wu X Zhuang W Xia L Chen Y Wu C. Tomato and lycopene and multiple health outcomes: umbrella review. Food Chem. (2021) 343:128396. 10.1016/j.foodchem.2020.128396 33131949

[16] Egger M Davey Smith G Schneider M Minder C. Bias in meta-analysis detected by a simple, graphical test. BMJ. (1997) 315:629–34. 10.1136/bmj.315.7109.629 9310563 PMC2127453

[17] Chen F Li Q Yu Y Yang W Shi F Qu Y. Association of vitamin C, vitamin D, vitamin E and risk of bladder cancer: a dose-response meta-analysis. Sci Rep. (2015) 5:9599. 10.1038/srep09599 25905583 PMC5386108

[18] Zhang D Xu P Li Y Wei B Yang S Zheng Y. Association of vitamin C intake with breast cancer risk and mortality: a meta-analysis of observational studies. Aging (Albany NY). (2020) 12:18415–35. 10.18632/aging.103769 32991322 PMC7585084

[19] Bandera EV Gifkins DM Moore DF McCullough ML Kushi LH. Antioxidant vitamins and the risk of endometrial cancer: a dose-response meta-analysis. Cancer Causes Control. (2009) 20:699–711. 10.1007/s10552-008-9283-x 19083131 PMC2772876

[20] Bai XY Qu X Jiang X Xu Z Yang Y Su Q. Association between dietary vitamin C intake and risk of prostate cancer: a meta-analysis involving 103,658 subjects. J Cancer. (2015) 6:913–21. 10.7150/jca.12162 26284143 PMC4532989

[21] Jia L Jia Q Shang Y Dong X Li L. Vitamin C intake and risk of renal cell carcinoma: a meta-analysis. Sci Rep. (2015) 5:17921. 10.1038/srep17921 26643589 PMC4672306

[22] Bo Y Lu Y Zhao Y Zhao E Yuan L Lu W. Association between dietary vitamin C intake and risk of esophageal cancer: a dose-response meta-analysis. Int J Cancer. (2016) 138:1843–50. 10.1002/ijc.29838 26355388

[23] Kong P Cai Q Geng Q Wang J Lan Y Zhan Y. Vitamin intake reduce the risk of gastric cancer: meta-analysis and systematic review of randomized and observational studies. PLoS ONE. (2014) 9:1–21. 10.1371/journal.pone.0116060 25549091 PMC4280145

[24] Fan H Kou J Han D Li P Zhang D Wu Q. Association between vitamin C intake and the risk of pancreatic cancer: a meta-analysis of observational studies. Sci Rep. (2015) 5:13973. 10.1038/srep13973 26360104 PMC4566085

[25] Heine-Bröring RC Winkels RM Renkema JM Kragt L van Orten-Luiten AC Tigchelaar EF. Dietary supplement use and colorectal cancer risk: a systematic review and meta-analyses of prospective cohort studies. Int J Cancer. (2015) 136:2388–401. 10.1002/ijc.29277 25335850

[26] Liu Y Yu Q Zhu Z Zhang J Chen M Tang P. Vitamin and multiple-vitamin supplement intake and incidence of colorectal cancer: a meta-analysis of cohort studies. Med Oncol. (2015) 32:434. 10.1007/s12032-014-0434-5 25491145

[27] Cao D Shen K Li Z Xu Y Wu D. Association between vitamin C Intake and the risk of cervical neoplasia: a meta-analysis. Nutr Cancer. (2016) 68:48–57. 10.1080/01635581.2016.1115101 26731169

[28] Zhou S Wang X Tan Y Qiu L Fang H Li W. Association between vitamin C intake and glioma risk: evidence from a meta-analysis. Neuroepidemiology. (2015) 44:39–44. 10.1159/000369814 25720916

[29] Luo J Shen L Zheng D. Association between vitamin C intake and lung cancer: a dose-response meta-analysis. Sci Rep. (2014) 4:6161. 10.1038/srep06161 25145261 PMC5381428

[30] Aune D Keum N Giovannucci E Fadnes LT Boffetta P Greenwood DC. Dietary intake and blood concentrations of antioxidants and the risk of cardiovascular disease, total cancer, and all-cause mortality: a systematic review and dose-response meta-analysis of prospective studies. Am J Clin Nutr. (2018) 108:1069–91. 10.1093/ajcn/nqy097 30475962 PMC6250988

[31] Psaltopoulou T Ntanasis-Stathopoulos I Tsilimigras DI Tzanninis IG Gavriatopoulou M Sergentanis TN. Micronutrient intake and risk of hematological malignancies in adults: a systematic review and meta-analysis of cohort studies. Nutr Cancer. (2018) 70:821–39. 10.1080/01635581.2018.1490444 30288994

[32] Mahdavi R Faramarzi E Seyedrezazadeh E Mohammad-Zadeh M Pourmoghaddam M. Evaluation of oxidative stress, antioxidant status and serum vitamin C levels in cancer patients. Biol Trace Elem Res. (2009) 130:1–6. 10.1007/s12011-008-8309-2 19148586

[33] Mastrangelo D Massai L Lo Coco F Noguera NI Borgia L Fioritoni G. Cytotoxic effects of high concentrations of sodium ascorbate on human myeloid cell lines. Ann Hematol. (2015) 94:1807–16. 10.1007/s00277-015-2464-2 26264692

[34] Chen Q Espey MG Krishna MC Mitchell JB Corpe CP Buettner GR. Pharmacologic ascorbic acid concentrations selectively kill cancer cells: action as a pro-drug to deliver hydrogen peroxide to tissues. Proc Natl Acad Sci U S A. (2005) 102:13604–9. 10.1073/pnas.0506390102 16157892 PMC1224653

[35] Chen Q Espey MG Sun AY Lee JH Krishna MC Shacter E. Ascorbate in pharmacologic concentrations selectively generates ascorbate radical and hydrogen peroxide in extracellular fluid in vivo. Proc Natl Acad Sci U S A. (2007) 104:8749–54. 10.1073/pnas.0702854104 17502596 PMC1885574

[36] Chen Q Espey MG Sun AY Pooput C Kirk KL Krishna MC. Pharmacologic doses of ascorbate act as a prooxidant and decrease growth of aggressive tumor xenografts in mice. Proc Natl Acad Sci U S A. (2008) 105:11105–9. 10.1073/pnas.0804226105 18678913 PMC2516281

[37] Pawlowska E Szczepanska J Blasiak J. Pro- and antioxidant effects of vitamin C in cancer in correspondence to its dietary and pharmacological concentrations. Oxid Med Cell Longev. (2019) 1–18. 10.1155/2019/7286737 31934267 PMC6942884

[38] Cameron E Pauling L Leibovitz B. Ascorbic acid and cancer: a review. Cancer Res. (1979) 39:663–81. 371790

[39] Lee B Oh SW Myung SK. Efficacy of vitamin C supplements in prevention of cancer: a meta-analysis of randomized controlled trials. Korean J Fam Med. (2015) 36:278–85. 10.4082/kjfm.2015.36.6.278 26634093 PMC4666862

[40] Dizdaroglu M Jaruga P. Mechanisms of free radical-induced damage to DNA. Free Radic Res. (2012) 46:382–419. 10.3109/10715762.2011.653969 22276778

[41] Pitocco D Zaccardi F Di Stasio E Romitelli F Santini SA Zuppi C. Oxidative stress, nitric oxide, and diabetes. Rev Diabet Stud. (2010) 7:15–25. 10.1900/RDS.2010.7.15 20703435 PMC2923377

[42] Lee KW Lee HJ Surh YJ Lee CY. Vitamin C and cancer chemoprevention: reappraisal. Am J Clin Nutr. (2003) 78:1074–8. 10.1093/ajcn/78.6.1074 14668266

[43] Han X Li J Brasky TM Xun P Stevens J White E. Antioxidant intake and pancreatic cancer risk: the Vitamins and Lifestyle (VITAL) Study. Cancer. (2013) 119:1314–20. 10.1002/cncr.27936 23280534 PMC3604041

[44] Sram RJ Binkova B Rossner P Jr. Vitamin C for DNA damage prevention. Mutat Res. (2012) 733:39–49. 10.1016/j.mrfmmm.2011.12.001 22178550

[45] Traber MG Stevens JF. Vitamins C and E: beneficial effects from a mechanistic perspective. Free Radic Biol Med. (2011) 51:1000–13. 10.1016/j.freeradbiomed.2011.05.017 21664268 PMC3156342

[46] Halliwell B. Vitamin C: antioxidant or pro-oxidant in vivo? Free Radic Res. (1996) 25:439–54. 10.3109/10715769609149066 8902542

[47] Leppert JT Shvarts O Kawaoka K Lieberman R Belldegrun AS Pantuck AJ. Prevention of bladder cancer: a review. Eur Urol. (2006) 49:226–34. 10.1016/j.eururo.2005.12.011 16413099

[48] Dusinska M Kazimirova A Barancokova M Beno M Smolkova B Horska A. Nutritional supplementation with antioxidants decreases chromosomal damage in humans. Mutagenesis. (2003) 18:371–6. 10.1093/mutage/geg002 12840111

[49] Federico A Morgillo F Tuccillo C Ciardiello F Loguercio C. Chronic inflammation and oxidative stress in human carcinogenesis. Int J Cancer. (2007) 121:2381–6. 10.1002/ijc.23192 17893868

[50] Cairns RA Harris IS Mak TW. Regulation of cancer cell metabolism. Nat Rev Cancer. (2011) 11:85–95. 10.1038/nrc2981 21258394

[51] Zhao F Wang M Li S Bai X Bi H Liu Y. DACH1 inhibits SNAI1-mediated epithelial–mesenchymal transition and represses breast carcinoma metastasis. Oncogenesis. (2015) 4:e143. 10.1038/oncsis.2015.3 25775416 PMC5399170

[52] Pathak SK Sharma RA Steward WP Mellon JK Griffiths TR Gescher AJ. Oxidative stress and cyclooxygenase activity in prostate carcinogenesis: targets for chemopreventive strategies. Eur J Cancer. (2005) 41:61–70. 10.1016/j.ejca.2004.09.028 15617991

[53] Kazmierczak-Baranska J Boguszewska K Adamus-Grabicka A Karwowski BT. Two faces of vitamin C-Antioxidative and pro-oxidative agent. Nutrients. (2020) 12:1–19. 10.3390/nu12051501 32455696 PMC7285147

[54] Knowles HJ Raval RR Harris AL Ratcliffe PJ. Effect of ascorbate on the activity of hypoxia-inducible factor in cancer cells. Cancer Res. (2003) 63:1764–8. Available online at: https://cancerres.aacrjournals.org/content/63/8/1764.long# 12702559

[55] Guz J Oliński R. The role of vitamin C in epigenetic regulation. Postepy Hig Med Dosw (Online). (2017) 71:747–60. 10.5604/01.3001.0010.3853 28894047

[56] Lian H Li WB Jin WL. The emerging insights into catalytic or non-catalytic roles of TET proteins in tumors and neural development. Oncotarget. (2016) 7:64512–25. 10.18632/oncotarget.11412 27557497 PMC5325459

[57] Lio CJ Yue X Lopez-Moyado IF Tahiliani M Aravind L Rao A. TET methylcytosine oxidases: new insights from a decade of research. J Biosci. (2020) 45:21. 10.1007/s12038-019-9973-4 31965999 PMC7216820

[58] Shekhawat J Gauba K Gupta S Choudhury B Purohit P Sharma P. Ten-eleven translocase: key regulator of the methylation landscape in cancer. J Cancer Res Clin Oncol. (2021) 147:1869–79. 10.1007/s00432-021-03641-3 33913031 PMC11802082

[59] Yin R Mao SQ Zhao B Chong Z Yang Y Zhao C. Ascorbic acid enhances Tet-mediated 5-methylcytosine oxidation and promotes DNA demethylation in mammals. J Am Chem Soc. (2013) 135:10396–403. 10.1021/ja4028346 23768208

[60] DiTroia SP Percharde M Guerquin MJ Wall E Collignon E Ebata KT. Maternal vitamin C regulates reprogramming of DNA methylation and germline development. Nature. (2019) 573:271–5. 10.1038/s41586-019-1536-1 31485074 PMC8423347

[61] Esteller M. Epigenetics in cancer. N Engl J Med. (2008) 358:1148–59. 10.1056/NEJMra072067 18337604

[62] Khan A Tania M Zhang D Chen H. Antioxidant enzymes and cancer. Chin J Cancer Res. (2010) 22:87–92. 10.1007/s11670-010-0087-7

[63] Sies H Jones DP. Reactive oxygen species (ROS) as pleiotropic physiological signalling agents. Nat Rev Mol Cell Biol. (2020) 21:363–83. 10.1038/s41580-020-0230-3 32231263

[64] DeBerardinis RJ Chandel NS. Fundamentals of cancer metabolism. Sci Adv. (2016) 2:e1600200. 10.1126/sciadv.1600200 27386546 PMC4928883

[65] Hoffer LJ Levine M Assouline S Melnychuk D Padayatty SJ Rosadiuk K. Phase I clinical trial of i. v ascorbic acid in advanced malignancy. Ann Oncol. (2008) 19:1969–74. 10.1093/annonc/mdn377 18544557

[66] Hussain SP Hofseth LJ Harris CC. Radical causes of cancer. Nat Rev Cancer. (2003) 3:276–85. 10.1038/nrc1046 12671666

[67] Buettner GR Moseley PL. EPR spin trapping of free radicals produced by bleomycin and ascorbate. Free Radic Res Commun. (1993) 19(Suppl 1): S89–93. 10.3109/10715769309056s89 7506694

[68] Bielski BH Rochter HW Chan PC. Some properties of the ascorbate free radical. Ann N Y Acad Sci. (1975) 258:231–7. 10.1111/j.1749-6632.1975.tb29283.x 942

[69] Lewis S. Vitamin C: Its Molecular Biology and Medical Potential. London: Academic Press (1976). p. 469–70.

[70] Cameron E Pauling L. The orthomolecular treatment of cancer. I the role of ascorbic acid in host resistance. Chem Biol Interact. (1974) 9:273–83. 10.1016/0009-2797(74)90018-0 4609626

[71] Cameron E Campbell A. The orthomolecular treatment of cancer. II Clinical trial of high-dose ascorbic acid supplements in advanced human cancer. Chem Biol Interact. (1974) 9:285–315. 10.1016/0009-2797(74)90019-2 4430016

[72] Institute of Medicine Panel on Micronutrients. Dietary Reference Intakes for Vitamin A, Vitamin K, Arsenic, Boron, Chromium, Copper, Iodine, Iron, Manganese, Molybdenum, Nickel, Silicon, Vanadium, and Zinc. Washington, DC: National Academies Press (2001). 25057538

[73] Carr AC Frei B. Toward a new recommended dietary allowance for vitamin C based on antioxidant and health effects in humans. Am J Clin Nutr. (1999) 69:1086–107. 10.1093/ajcn/69.6.1086 10357726

