# Supplementary material for: Vitamin C Intake and Cancers: An Umbrella Review
Source: Front Nutr. 2022 Jan 20;8:812394. doi: 10.3389/fnut.2021.812394 (PMC8812486; doi:10.3389/fnut.2021.812394)
Supplement: Supplementary file 1 [file Table_1.DOCX]

Supplementary Table 1. Assessments of AMSTAR scores for studies including Vitamin C.

| Outcome | Assessed with | Author | Year | A priori design provided | Duplicate study selection & data extraction | At least two electronic databases searched | Status of  publication used as an inclusion criterion | List of  included and excluded studies provided | Characteristics of included  studies provided | Scientific quality of  included studies assessed | Scientific quality of the included studies used  appropriately to form  conclusions | Appropriate methods to  combine studies | Publication bias assessed | Conflict of interest included | Total AMSTAR Score |
| --- | --- | --- | --- | --- | --- | --- | --- | --- | --- | --- | --- | --- | --- | --- | --- |
| Bladder cancer risk | Highest versus lowest | Chen et al. | 2015 | 0 | 0 | 1 | 0 | 0 | 1 | 0 | 0 | 1 | 1 | 1 | 5 |
| Breast cancer risk | Highest versus lowest | Zhang et al. | 2020 | 0 | 1 | 1 | 0 | 1 | 1 | 1 | 0 | 1 | 1 | 1 | 8 |
| Breast cancer specific mortality | Highest versus lowest | Zhang et al. | 2020 | 0 | 1 | 1 | 0 | 1 | 1 | 1 | 0 | 1 | 0 | 1 | 7 |
| Breat cancer recurrence | Highest versus lowest | Zhang et al. | 2020 | 0 | 1 | 1 | 0 | 1 | 1 | 1 | 0 | 1 | 0 | 1 | 7 |
| Breat cancer all cause mortality | Highest versus lowest | Zhang et al. | 2020 | 0 | 1 | 1 | 0 | 1 | 1 | 1 | 0 | 1 | 0 | 1 | 7 |
| Cervical neoplasm | 50 mg/d increment | Cao et al. | 2016 | 0 | 1 | 1 | 0 | 1 | 1 | 1 | 0 | 1 | 1 | 0 | 7 |
| Colon Cancer risk | Use versus no use | Heine-Bröring et al. | 2015 | 0 | 1 | 1 | 0 | 1 | 1 | 0 | 0 | 1 | 0 | 1 | 6 |
| Colorectal cancer risk | highest versus lowest | Liu et al. | 2014 | 0 | 1 | 0 | 0 | 1 | 1 | 1 | 0 | 1 | 1 | 1 | 7 |
| Endometrial cancer | 50 mg/1,000 kcal | Bandera et al. | 2008 | 0 | 1 | 1 | 0 | 0 | 1 | 0 | 0 | 1 | 0 | 0 | 4 |
| Esophageal cancer risk | highest versus lowest | Bo et al. | 2015 | 0 | 1 | 1 | 0 | 1 | 1 | 0 | 0 | 1 | 1 | 0 | 6 |
| Gastric cancer risk | highest versus lowest | Kong et al. | 2014 | 0 | 1 | 1 | 0 | 1 | 1 | 1 | 0 | 1 | 0 | 1 | 7 |
| Glioma risk | highest versus lowest | Zhou et al. | 2015 | 0 | 1 | 1 | 0 | 1 | 1 | 0 | 0 | 1 | 1 | 0 | 6 |
| non-Hodgkin lymphoma risk | highest versus lowest | Psaltopoulou et al. | 2018 | 0 | 1 | 0 | 0 | 1 | 1 | 1 | 0 | 1 | 1 | 1 | 7 |
| Lung cancer risk | highest versus lowest | Luo et al. | 2014 | 0 | 1 | 1 | 0 | 1 | 1 | 0 | 0 | 1 | 1 | 1 | 7 |
| Ovarian cancer risk | highest versus lowest | Long et al. | 2020 | 0 | 1 | 1 | 0 | 1 | 1 | 0 | 0 | 1 | 1 | 1 | 7 |
| Pancreatic cancer risk | highest versus lowest | Fan et al. | 2015 | 0 | 1 | 1 | 0 | 1 | 1 | 1 | 0 | 1 | 1 | 1 | 8 |
| Prostate cancer risk | highest versus lowest | Bai et al. | 2015 | 0 | 1 | 1 | 0 | 1 | 1 | 1 | 0 | 1 | 1 | 1 | 8 |
| Renal cell cancer risk | highest versus lowest | Jia et al. | 2015 | 0 | 1 | 1 | 0 | 0 | 1 | 0 | 0 | 1 | 1 | 1 | 6 |
| Total cancer risk | highest versus lowest | Aune et al. | 2018 | 0 | 1 | 1 | 0 | 1 | 1 | 1 | 0 | 1 | 1 | 1 | 8 |

Supplementary Table 2. GRADE classification of quality of evidence for Vitamin C consumption and cancer outcomes.

| Outcome | Categories | Assessed with | Author | Year | No. of  studies | Cohort | Case-control | Risk of  bias | Inconsistency | Indirectness | Imprecision | Publication bias | Plausible confounding | Magnitude of  effect | Dose-response gradient | Quality |
| --- | --- | --- | --- | --- | --- | --- | --- | --- | --- | --- | --- | --- | --- | --- | --- | --- |
| Bladder cancer risk | Supplementary+dietary | Highest versus lowest | Chen et al. | 2015 | 8 | 3 | 5 | serious risk | serious  inconsistency | no serious  indirectness | no serious  imprecision | strongly suspected | Could reduce effect | no | no | low |
| Bladder cancer risk | supplementary | Highest versus lowest | Chen et al. | 2015 | 9 | 6 | 3 | serious risk | serious  inconsistency | no serious  indirectness | no serious  imprecision | strongly suspected | Could reduce effect | no | no | low |
| Bladder cancer risk | Dietary | Highest versus lowest | Chen et al. | 2015 | 14 | 7 | 7 | serious risk | serious  inconsistency | no serious  indirectness | no serious  imprecision | NA | Could reduce effect | no | no | moderate |
| Breast cancer risk | Dietary | Highest versus lowest | Zhang et al. | 2020 | 31 | 15 | 16 | serious risk | serious  inconsistency | no serious  indirectness | no serious  imprecision | strongly suspected | Would not reduce effect | no | Yes | low |
| Breast cancer risk | supplementary | Highest versus lowest | Zhang et al. | 2020 | 13 | 9 | 4 | serious risk | No serious  inconsistency | no serious  indirectness | no serious  imprecision | strongly suspected | Would not reduce effect | no | Yes | moderate |
| Breast cancer specific mortality | NA | Highest versus lowest | Zhang et al. | 2020 | 6 | 5 | 1 | serious risk | No serious  inconsistency | no serious  indirectness | no serious  imprecision | NA | Would not reduce effect | no | no | moderate |
| Breast cancer recurrence | NA | Highest versus lowest | Zhang et al. | 2020 | 2 | 2 | 0 | serious risk | No serious  inconsistency | no serious  indirectness | no serious  imprecision | NA | Would not reduce effect | no | no | moderate |
| Breast cancer all-cause mortality | NA | Highest versus lowest | Zhang et al. | 2020 | 7 | 7 | 0 | serious risk | No serious  inconsistency | no serious  indirectness | no serious  imprecision | NA | Would not reduce effect | no | no | moderate |
| Cervical neoplasm risk | NA | 50 mg/d increment | Cao et al. | 2016 | 12 | 1 | 11 | serious risk | serious  inconsistency | no serious  indirectness | no serious  imprecision | strongly suspected | Would not reduce effect | no | Yes | low |
| Colon Cancer risk | Dietary | Use versus no use | Heine-Bröring et al | 2014 | 3 | 3 | 0 | serious risk | serious  inconsistency | no serious  indirectness | no serious  imprecision | NA | Would not reduce effect | no | Yes | moderate |
| Colorectal cancer risk | NA | highest versus lowest | Liu et al. | 2015 | 13 | 13 | 0 | serious risk | No serious  inconsistency | no serious  indirectness | no serious  imprecision | Not detected | Could reduce effect | no | no | high |
| Endometrial cancer risk | NA | 50 mg/1,000 kcal | Badera et al. | 2008 | 11 | 1 | 10 | serious risk | serious  inconsistency | no serious  indirectness | no serious  imprecision | NA | Would not reduce effect | no | Yes | moderate |
| Esophageal cancer risk | Dietary | highest versus lowest | Bo et al. | 2015 | 20 | 1 | 19 | serious risk | serious  inconsistency | no serious  indirectness | no serious  imprecision | Not detected | Would not reduce effect | no | Yes | moderate |
| Gastric cancer risk | Dietary | highest versus lowest | Kong et al. | 2014 | 37 | 3 | 34 | serious risk | No serious  inconsistency | no serious  indirectness | no serious  imprecision | Not detected | Could reduce effect | No | Yes | moderate |
| Glioma risk | NA | highest versus lowest | Zhou et al. | 2015 | 15 | 2 | 13 | serious risk | No serious  inconsistency | no serious  indirectness | no serious  imprecision | Not detected | Would not reduce effect | no | no | moderate |
| non-Hodgkin lymphoma risk | Supplementary | highest versus lowest | Psaltopoulou et al. | 2018 | 8 | 8 | 0 | serious risk | No serious  inconsistency | no serious  indirectness | no serious  imprecision | NA | Would not reduce effect | No | No | moderate |
| Lung cancer risk | NA | highest versus lowest | Luo et al. | 2014 | 21 | 11 | 10 | serious risk | serious  inconsistency | no serious  indirectness | no serious  imprecision | Not detected | Would not reduce effect | No | Yes | moderate |
| Pancreatic cancer risk | NA | highest versus lowest | Fan et al. | 2015 | 17 | 4 | 13 | serious risk | serious  inconsistency | no serious  indirectness | no serious  imprecision | Not detected | Would not reduce effect | No | No | low |
| Prostate cancer risk | Dietary | highest versus lowest | Bai et al. | 2015 | 18 | 6 | 12 | serious risk | serious  inconsistency | no serious  indirectness | no serious  imprecision | strongly suspected | Would not reduce effect | No | Yes | low |
| Renal cell cancer risk | NA | highest versus lowest | Jia et al. | 2015 | 10 | 3 | 7 | serious risk | No serious  inconsistency | no serious  indirectness | no serious  imprecision | Not detected | Would not reduce effect | No | No | moderate |
| Ovarian cancer risk | NA | highest versus lowest | Long et al. | 2020 | 16 | 5 | 11 | serious risk | serious  inconsistency | no serious  indirectness | no serious  imprecision | Not detected | Would not reduce effect | Yes | no | moderate |
| Total cancer risk | Dietary | highest versus lowest | Aune et al. | 2018 | 7 | 7 | 0 | serious risk | No serious  inconsistency |  | no serious  imprecision | Not detected | Would not reduce effect | No | Yes | high |
